# Promotion of HepG2 cell apoptosis by flower of *Allium atroviolaceum* and the mechanism of action

**DOI:** 10.1186/s12906-017-1594-6

**Published:** 2017-02-10

**Authors:** S. Khazaei, R. Abdul Hamid, N. Mohd Esa, V. Ramachandran, Ghomi Tabatabaee F. Aalam, A. Etemad, P. Ismail

**Affiliations:** 1 Department of Biomedical Science, Faculty of Medicine and Health Sciences, Universiti Putra Malaysia, Serdang, Selangor 43400 Malaysia; 2 Department of Nutrition and Dietetics, Faculty of Medicine and Health Sciences, Universiti Putra Malaysia, Serdang, Selangor 43400 Malaysia; 3 Malaysian Research Institute of Aging, Universiti Putra Malaysia, Serdang, Selangor 43400 Malaysia

**Keywords:** *Allium atroviolaceum*, HepG2, Apoptosis

## Abstract

**Background:**

Liver cancer is a high incidence and fatal disease, the fifth most frequent cancer worldwide that is usually diagnosed at an advanced stage. The number of deaths from liver cancer has not declined even following various therapies. Plant secondary metabolites and their semi-synthetic derivatives play a principal role in anti-cancer drug therapy, since they are effective in the treatment of specific characteristics while also reducing side effects. *Allium atroviolaceum*, a plant of the genus *Allium* has been used in folk medicine to protect against several diseases. However, cytotoxicity and the anti-proliferative effect of *Allium atroviolaceum* remain unclear. This work aims to investigate the anticancer properties of *Allium atroviolaceum* and the mechanism of action.

**Methods:**

To evaluate the *in vitro* cytotoxicity of flower of *Allium atroviolaceum*, methanol extract at a dose range from 100 to 3.12 μg/ml was assessed against the HepG2 hepatocarcinoma cell line, and also on normal 3T3 cells, by monitoring proliferation using the MTT assay method. A microscopy study was undertaken to observe morphological changes of HepG2 cells after treatment and cell cycle arrest and apoptosis were studied using flow cytometry. The apoptosis mechanism of action was assessed by the level of caspase-3 activity and expression of apoptosis related genes, *Bcl-2*, *Cdk1* and *p53*. The combination effect of the methanolic extract with doxorubicin was also investigated by determination of a combination index.

**Results:**

The results demonstrated growth inhibition of cells in both dose- and time-dependent manners, while no cytotoxic effect on normal cell 3T3 was found. The results revealed the occurrence of apoptosis, illustrated by sub-G0 cell cycle arrest, the change in morphological feature and annexin-V and propidium iodide staining, which is correlated with *Bcl-2* downregulation and caspase-3 activity, but *p53*-independent. In addition, a combination of *Allium atroviolaceum* and doxorubicin led to a significant synergistic effect.

**Conclusion:**

These findings suggest that *Allium atroviolaceum* flower extract has potential as a potent cytotoxic agent against HepG2 cell lines, as it has commendable anti-proliferative activities against human hepatocarcinoma and it can be considered as an effective adjuvant therapeutic agent after the clinical trials.

## Background

Cancer, as a complex disease that results from genetic and epigenetic modifications of tumour suppressor genes or oncogenes, can be developed because of alterations of apoptosis-signalling pathways. Breakdown of the apoptosis process is observed in many human tumours, which may lead to transformation of a normal cell to a tumour cell [1]. Apoptosis is one of the primary targets for most conventional anti-cancer drugs. The drugs are able to induce fatal intracellular damage, which often activates a down-stream cascade of molecular events [2]. Hepatocellular carcinoma (HCC) is a high incidence and fatal disease that is usually diagnosed at an advanced stage [3], the fifth most frequent cancer worldwide [4] and the third most fatal cancer [5]. In 2008, 748,000 liver cancer cases and 696,000 mortalities were estimated worldwide [6]. In Asian regions, HCC is the second most mortal cancer [7] with a peak incidence in East Asia [8]. Despite recent scientific advancement in hepatology, liver problems continue to increase [9]. There is no beneficial cure for this malignancy [10] and the recovery rate of HCC is low in most cases [11]. In addition, the inevitable side effects, such as toxicity to normal cells and bone marrow illustrate an instant demand to search for better methods and novel anti-cancer agents that would decrease the mortality rate of HCC with fewer side effects [12].

Natural products or their derivatives and synthetic pharmaceuticals based on natural product models are defined as natural origin [13] and play a principal role in anti-cancer drug therapy [14]. Natural products as medicines are effective in the treatment of specific characteristics while also reducing side effects [15]. Taken together, research in the field of natural products is in high demand to help humans overcome many newly emerging and known diseases, particularly cancers.

*Allium atroviolaceum* (A. atroviolaceum) is one of the lesser known species of *Allium.* The medicinal potency of the species of the *Allium* genus indicates tumour inhibitory effects at several stages of carcinogenesis, resulting from the high content of flavonols and organosulfur compounds; however, the mechanisms of action remain unclear [16]. Study of some species of *Allium* revealed different levels of anti-growth activity on the cancer cell lines; and minor cytotoxicity against the normal cell line [17] which makes this genus valuable for anticancer study.

The pharmaceutical value of *A. atroviolaceum* remains undiscovered. However, analysis of a flower extract has led to the isolation of a new sapogenin, named atroviolacegenin, a rare feature among sapogenins and saponins [18]. Saponins are natural glycosides which possess a wide range of pharmacological properties including cytotoxic activity [19]. Moreover, an investigation of the *A. atroviolaceum* chemical composition revealed a significantly high percentage of phenolic and organosulfur compounds [20]. Nowadays, inhibition of cancer cell growth by biosulfur compounds derived from *Allium*s has been the topic of intense research. They are efficient to alter carcinogen metabolism [21]. Study of the anticancer effect of *A. atroviolaceum* and understanding of its effects at a molecular level may lead to an effective cancer treatment and a promising approach to control of cancer.

In the current study, we hypothesize that flower extract of *A. atroviolaceum* exhibits cytotoxic activity against liver tumour cells, including a selective cytostatic effect that potentiates use as an anti-cancer drug. Furthermore, the extract may contain multiple bioactive compounds that could work alone or in combination to restrict cell survival.

## Methods

### Plant material

The plant sample was collected from Mazandaran, Iran in June, 2013. The plant sample was identified by Dr. Bahman Eslami (Assistant Professor of Plant Systems, Islamic Azad University of Ghaemshahr, Iran); the voucher specimens were deposited in Islamic Azad University of Ghaemshahr, Iran (No 720-722). Fresh flower of *A. atroviolaceum* (FAA) was collected, washed and air dried at room temperature. The dried material was homogenized to obtain a coarse powder and stored in airtight bottles. Approximately 5 gm of the powdered material was subjected to soxhlet (Electrothermal Eng., Rochford, UK) extraction using 150 ml 70% methanol. The extract was concentrated under reduced pressure by rotary evaporator (Büchi Labortechnik AG, Flawil, Switzerland) and solidified by freeze drier (SP Scientific, NY, USA) [22]. The dry residue of methanol extract was dissolved in dimethyl sulfoxide (DMSO) (Sigma-Aldrich, MO, USA) to obtain the stock solution (1000 μg/ml).

### Cell culture

Human hepatoma HepG2 cells and mouse normal embryo cells (3T3) were obtained from the American Type Culture Collection (VA, USA). The cells were grown in RPMI-1640 supplemented with 10% FBS and 100 IU/ml penicillin streptomycin. The cultures were maintained at 37 °C in a humidified atmosphere of 5% CO_2_.

### MTT Cytotoxicity assay

HepG2 and normal 3 T3 cells were seeded at a density of 1 × 10^6^/well into 96-well culture plates, and incubated overnight before being exposed to various concentrations of FAA extract (100, 50, 25, 12.5, 6.25 and 3.12 μg/ml). Doxorubicin was used as the positive control and untreated media was the negative control. After 24, 48 and 72 h, 20 ug/ml of MTT solution was added to each well and incubated for 4 h. Each time course study was repeated at least three times. After addition of 100 μl of DMSO, the absorbance was measured with an ELISA reader (BMG Labtech, Ortenberg, Germany) at a test wavelength of 540 nm and a reference wavelength of 690 nm. The absorbance of the treated and control cells were used to determine the cytotoxicity of extract according to the following formula:

Cytotoxicity (%) = Absorbance of treated cells/absorbance of negative control × 100 [23].

### Microscopic examination

HepG2 cells were cultured into a six-well plate (1 × 10^6^ cell/ml) and after being treated with IC_50_ concentration of FAA, morphological apoptotic changes were examined after 24, 48 and 72 h incubation and photographed using a phase-contrast microscope (Olympus Corporation, Tokyo, Japan) [24].

### Acridine orange/propidium iodide (AO/PI) double staining

Acridine orange/propidium iodide (AO/PI) double staining was used to observe the changes of apoptotic cell nuclei. When AO passes through the complete cell membrane, the nuclear DNA appears in green fluorescence while PI emits a red–orange fluorescence in the nuclear DNA of damaged cells [25]. The cells were seeded at a density of 1 × 10^6^ cells per well of six-well plate and after incubation for 24 h, the old media were replaced with the media treated with IC_50_ of FAA. After 24, 48 and 72 h, the cells were washed with PBS. The mixture of 10 μg/ml acridine orange and 10 μg/ml propidium iodide (dissolved in PBS) was added to HepG2-treated cells and then immediately observed under Leica fluorescence microscope DM 2500 (Leica Microsystem, Wetzlar, Germany) with 100x magnification. Images were captured using an Alpha Imager (AlphaInnotech, CA, USA). Each experiment was assayed three times (*n* = 3) [26].

### Cell cycle analysis

Cell cycle analysis was carried out using a flow cytometer. Briefly, after plating 1× 10^6^ cells on a 25 cm^2^ culture flask for 24 h, cells were incubated with the concentration of FAA that induced 25, 50 and 75% growth inhibition (IC_25_, IC_50_ and IC_75_) for 24, 48 and 72 h. Thereafter, cells were trypsinized, centrifuged at 1000 rpm for 10 minutes, washed with PBS and fixed with 70% ethanol overnight at 20 °C. The fixed cells were washed with PBS and incubated with 500 μl PI/RNase (400 μl propidium iodide and 100 μl ribonuclease A). Stained cells were incubated at room temperature in the dark for 30 min before analysis. Cell-cycle distribution was then analysed by flow cytometry using the BD LSRFortessa™ Cell Analyzer (Becton Dickinson, NJ, USA). The cell cycle distribution of 10,000 cells was recorded and the percentage of cells at G0/G1, S, and G2/M phases was analysed with BD FACSDiva™ software [27].

### Flow cytometric analysis of apoptosis using Annexin V

An annexin V apoptosis detection kit for flow cytometry (Sigma-Aldrich, MO, USA) was used. The annexin V assay was carried out in conjunction with PI staining. HepG2 cells were cultured for 24 h in a 25 cm^2^ culture flask (1 × 10^6^ cells/well) in the presence of different concentrations of the extract (IC_25_, IC_50_ and IC_75_). After 24, 48 and 72 h, cells were harvested by trypsinization and centrifugation at 1000 rpm for 5 min and then re-suspended in 1x binding buffer prior to staining with 5 μl of annexin V and 10 μl of propidium iodide solution for 10 min at room temperature. FACS analysis was then carried out according to the manufacturer’s instructions, using a BD LSRFortessa™ Cell Analyzer (Becton Dickinson, NJ, USA). About 10,000 counts were recorded in each analysis [28].

### Caspase-3 colorimetric assay

A caspase colorimetric assay kit (Biovision, CA, USA) was used to measure the caspase -3 activity in treated cell line according to manufacturer’s instructions. The cells (10^6^/ml) were placed in a six-well plate for 24 h before treatment with various concentrations of FAA. After 24, 48 and 72 h, treated cells were collected into micro-centrifuge tubes and centrifuged at 1000 rpm for 5 min. Following two washes of pelleted cells with PBS, lysis buffer (50 μl) was added and mixed well. The cells were then incubated on ice for 10 min and subjected to centrifugation at 10,000 rpm for 1 min. Supernatants (50 μl) were transferred into wells of a 96-well plate; 50 ml of 2 × reaction buffer containing 0.5 μl DTT and 5 ml of caspases substrate was added to each sample. Samples were then incubated at 37 °C for 2 h in the dark. The cleavage of labelled substrate pNA into chromo-phore p-nitroanilide (pNA) was determined by measuring the absorbance (optical density, OD) at 405 nm using a FLUOstar Omega microplate reader (BMG Labtech, Ortenberg, Germany). The result of the induced group’s caspases activity was obtained by computing OD inducer/OD negative control with the background OD values from cell lysates and buffers subtracted [23].

### Quantitative polymerase chain reaction (qPCR)

To examine the expression of the target genes in HepG2 cell lines, RNA extraction including DNase treatment was carried out by RNeasy mini kit (Qiagen Inc., CA, USA), according to the manufacturers protocol. RNA concentration was measured using a Thermo Scientific NanoDrop™ 1000 Spectrophotometer along with its analytical software V3.7 (Thermo Fisher Scientific, DE, USA), RNA samples without indication of degradation were further assessed on a Bioanalyzer (Agilent 2100 Bioanalyzer™ system-Agilent Technologies, Waldbronn, Germany). Total RNA (1 μl) was reverse-transcribed using an RT2 first strand kit (Qiagen Inc., CA, USA). Quantitative PCR was performed on reference and target genes. The gene primer (RT^2^ qPCR Primer Assays, Qiagen Inc., CA, USA) sequences were selected based on previous databases and on publications reporting stable gene expression profiles. The respective forward and reverse primers are as follows: *Bcl-2*: 5’-TAC CTG AAC CGG CAC CTG-3’ and 5’- GCC GTA CAG TTC CAC AAA GG-3’; *Cdk1*: 5’- GGGTCAGCTCGCTACTCAAC-3’ and 5’-AAGTTTTTGACGTGGGATGC-3’; *p53*: 5’- TGT GGA GTA TTT GGA TGA CA-3’ and 5’- GAA CAT GAG TTT TTT ATG GC-3’; GAPDH: 5--TCCTGCACCA CCAACTGCTTAG-3’ and 5’- GGCATGGACTGTGG TCATGAGT-3’.

A sample without cDNA template (ntc) was used as the negative control. qPCR was performed on the Corbett Rotor-Gene 6000 (Qiagen Inc., CA, USA). RNase/DNase-free water (10.5 μL), RT2 SYBR® green master mix (12.5 μL), assay primer (1 μL) and cDNA template was mixed to a final volume of 25 μl and run at 95 °C for 10 minutes to activate the enzyme, 40 cycles of 15 seconds at 95 °C (denaturation) followed by 30 seconds at 60 °C (annealing and synthesis). The Ct cycle was used to determine the expression level of control and FAA treated cells. The gene expression level was then calculated by the following formula:

2^ΔΔCt^ = 2^Ct(treated cells) – Ct (control cells)^ [29].

### Determination of combination index

HepG2 cells were seeded at 10^6^ cells per ml, allowed to attach overnight and treated with the FAA, doxorubicin or their combination for 24, 48 and 72 h at 37 °C. The cytotoxic effect was determined using MTT assay. The IC_50_ obtained for single-agents were compared to the IC_50_ calculated for each cytotoxic agent after combination of two drugs. The combined effect of FAA and doxorubicin was then analysed using the CompuSyn software in different concentrations. All the tests were performed in triplicate. The CI value <1, =1 and >1 represent the synergistic, additive and antagonistic effects, respectively [30].

### Statistical analysis

The data were expressed as means ± SD, and significant differences were determined by one-way analysis of variance (ANOVA) followed by Duncan’s multiple range tests and Student's *t*-test. A *p*-value of less than 0.05 was considered statistically significant.

## Results

### Optimum dose of FAA extract to inhibit HepG2 cell proliferation

**Fig. 1:**
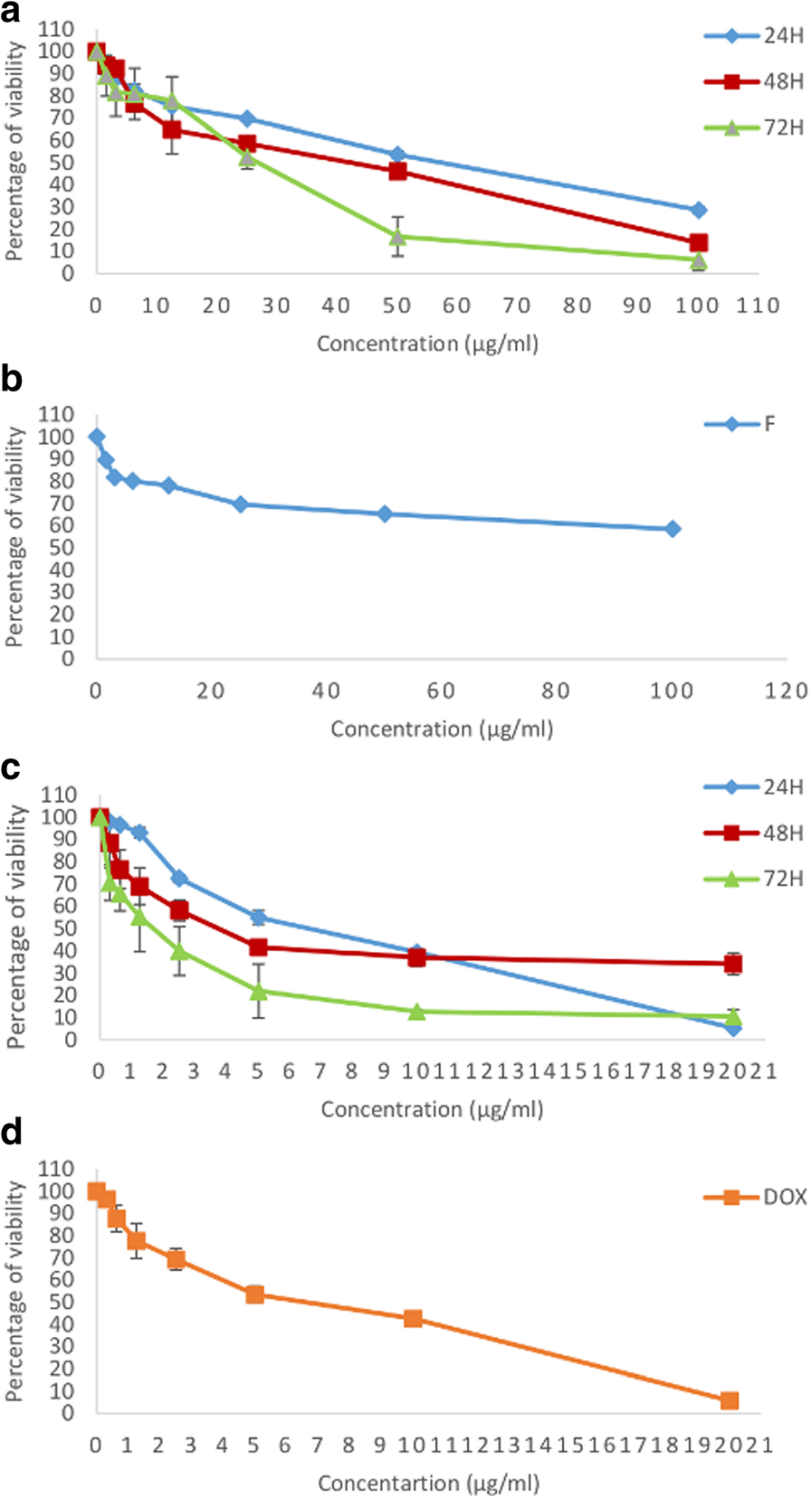
Antiproliferatic activity of FAA and doxorubicin on HepG2 and normal 3T3 cells. The effect of FAA on proliferation of (**a**) HepG2 and (**b**) 3T3 cells and doxorubicin on proliferation of (**c**) HepG2 [31] and (**d**) 3T3 cells. Values are means ± SD of three independent experiments

The anti-proliferative activities of methanol extract from FAA illustrated an inhibition effect on the cell proliferation in a time and dose-dependent manner. HepG2 cells treated with FAA showed inhibited cell proliferation at 24 h with the IC_50_ value of 57.5 ± 4.95 μg/ml, which markedly decreased to 44 μg/ml at 48 h and 26.67 ± 3.5 μg/ml at 72 h (Fig. 1a). Moreover, according to our previous study, doxorubicin was considerably more toxic against HepG2 cells whose IC_50_ was 4.75 ± 0.6 at 24 h, which gradually decreased to 3.47 ± 0.3 at 48 h and 1.7 ± 1 at 72 h (Fig. 1c) [31]. However, doxorubicin’s toxicity towards healthy mouse fibroblast cells, 3T3, was also considerably higher than FAA, which inhibits 50% cell viability at 6.45 ± 1.3 μg/ml (Fig. 1d) while FAA showed selective cytotoxicity, as the CC_50_ of normal cell is > 100 μl of FAA (Fig. 1b).

### Microscopic evaluation of morphological changes in HepG2 cells

**Fig. 2:**
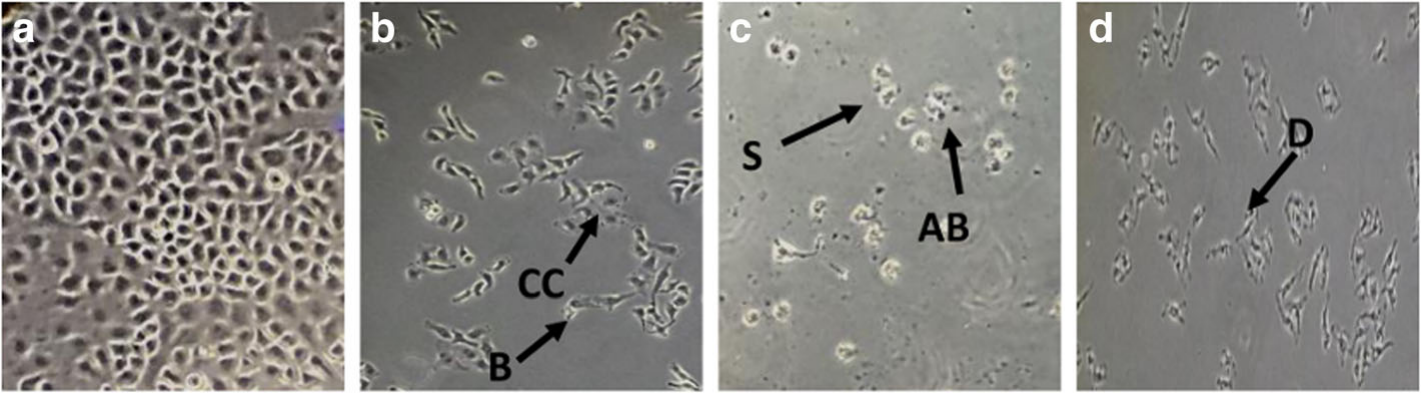
Representative images to show morphological observation of HepG2 No treatment (**a**), treatment with FAA for 24 h (**b**), 48 h (**c**), and 72 h (**d**) observed under inverted light microscopy (40X). Live cells (L), cytoplasm condensation (CC), blebbing (B), shrinkage (S), apoptotic bodies (AB) and debris (D). Similar cellular morphology was observed in three independent experiments (*n* = 3)

Morphological changes in the cells and the nuclei were observed under 40X magnification of the inverted phase contrast microscope, aided by AO/PI staining after treatment for 24, 48 and 72 h. Phase contrast microscopy of the cells revealed the original morphological form of control cells, most of which were adherent to the surface, but the presence of floating or detachment of non-viable cells in a dose- and time-dependent manner. Exposure of the cancer cells to FAA led to cytoplasm condensation (24 h), shrinkage and formation of apoptotic bodies (48 h) and the formation of debris (72 h) that are classic morphologies of apoptosis [32]. There was a visible loss of contact and rounding of cell shape post-treatment as compared to the tightly packed and distinctively epithelial monolayer formation in the untreated cells, indicative of apoptosis (Fig. 2).

**Fig. 3:**
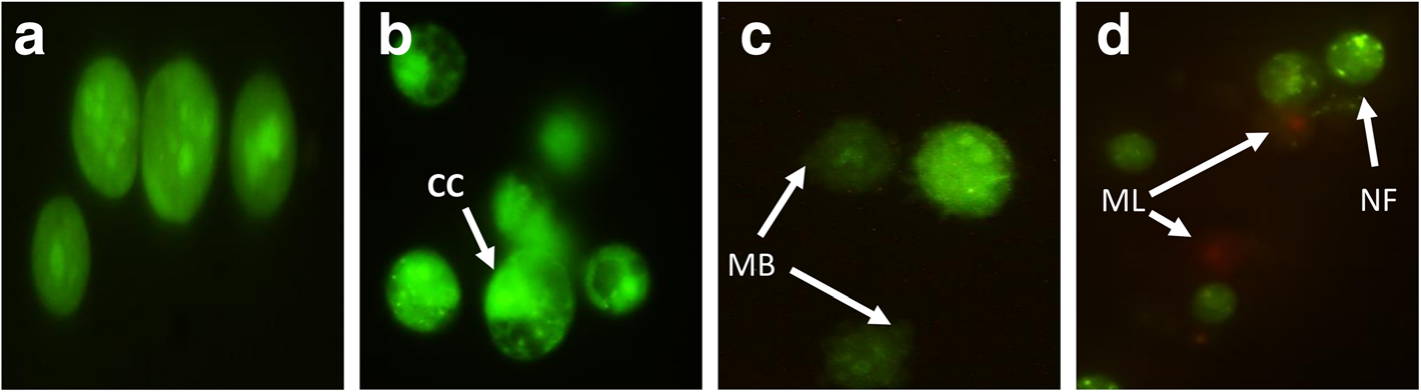
Treated HepG2 stained with AO/PI observed under fluorescence microscope. Photographic documentation was carried out at 40x magnification. **a** Control (untreated) cells (**b**) Cell treated with FAA at 24 h (**c**) 48 h (**d**) 72 h. Treated cells showed the typical characteristic of apoptosis such as nuclear margination (NM), chromatin condensation (CC), nuclear fragmentation (NF), membrane blebbing (MB), and membrane loss (ML)

In order to aid the visualization, cells were stained with AO/PI mixture and nuclear morphology changes were observed under the fluorescence microscope. The cells treated with FAA showed nuclear margination and chromatin condensation (24 h), membrane blebbing (48 h), nuclear fragmentation and membrane loss (72 h). Cells stained with orange colour indicated loss of cell membrane integrity. Morphological damage was seen in cell lines when treated with FAA as compared to undamaged nuclei in untreated cells. The untreated cells were live and stained bright green (Fig. 3).

### Effect of FAA extract on cell cycle phase distribution

**Fig. 4:**
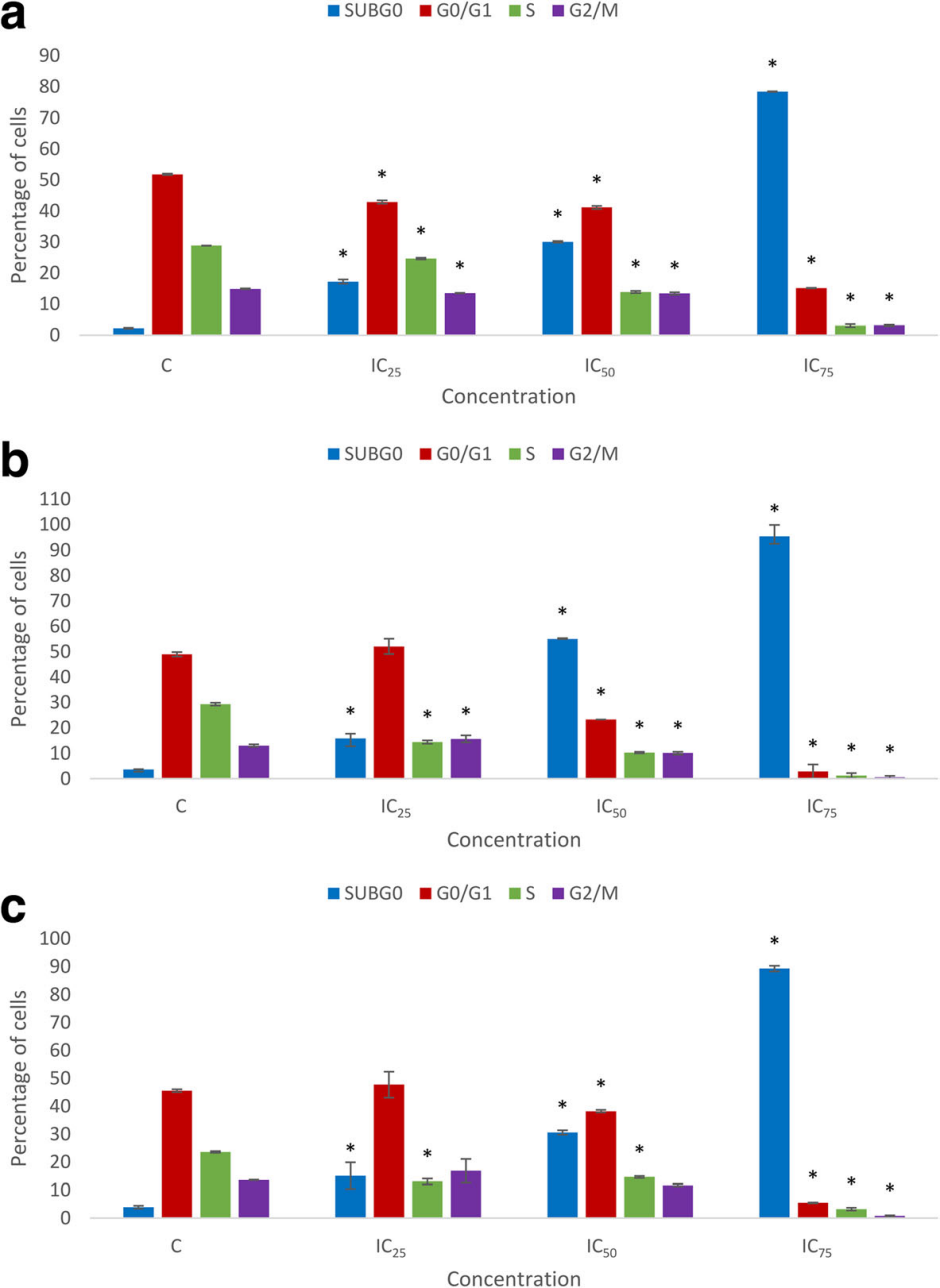
Variation in the percentage of HepG2 cells present in each phase of the cell cycle in time and dose course. Comparison between untreated cells and cells exposed to IC_25_ = 42, IC_50_ = 26.67 and IC_75_ = 11.67 of FAA for (**a**) 24, (**b**) 48 and (**c**) 72 h. Values are presented as means (*n* = 3) ± S.E. *signified (*p* < 0.05)

Cell cycle distribution of FAA-treated cells in a concentration- and time-response manner was assessed by flow cytometry. Synchronized cells exposed to FAA indicated a noticeable enhancement in cell proportion in sub-G0 phase in a time- and dose-dependent manner. In addition, the cells treated with IC_25_ concentration of FAA showed a significant increase from 12.9% (in the control cells) to 15.56% in the G2/M phase at 48 h. These results indicate that the anti-proliferative effect of FAA is not associated with cell cycle arrest, except in low concentration of FAA at 48 h where the anti-proliferative effect might be related to an arrest in G2/M phase of the cell cycle (Fig. 4).

### FAA extract induces apoptosis in HepG2 cells

**Fig. 5:**
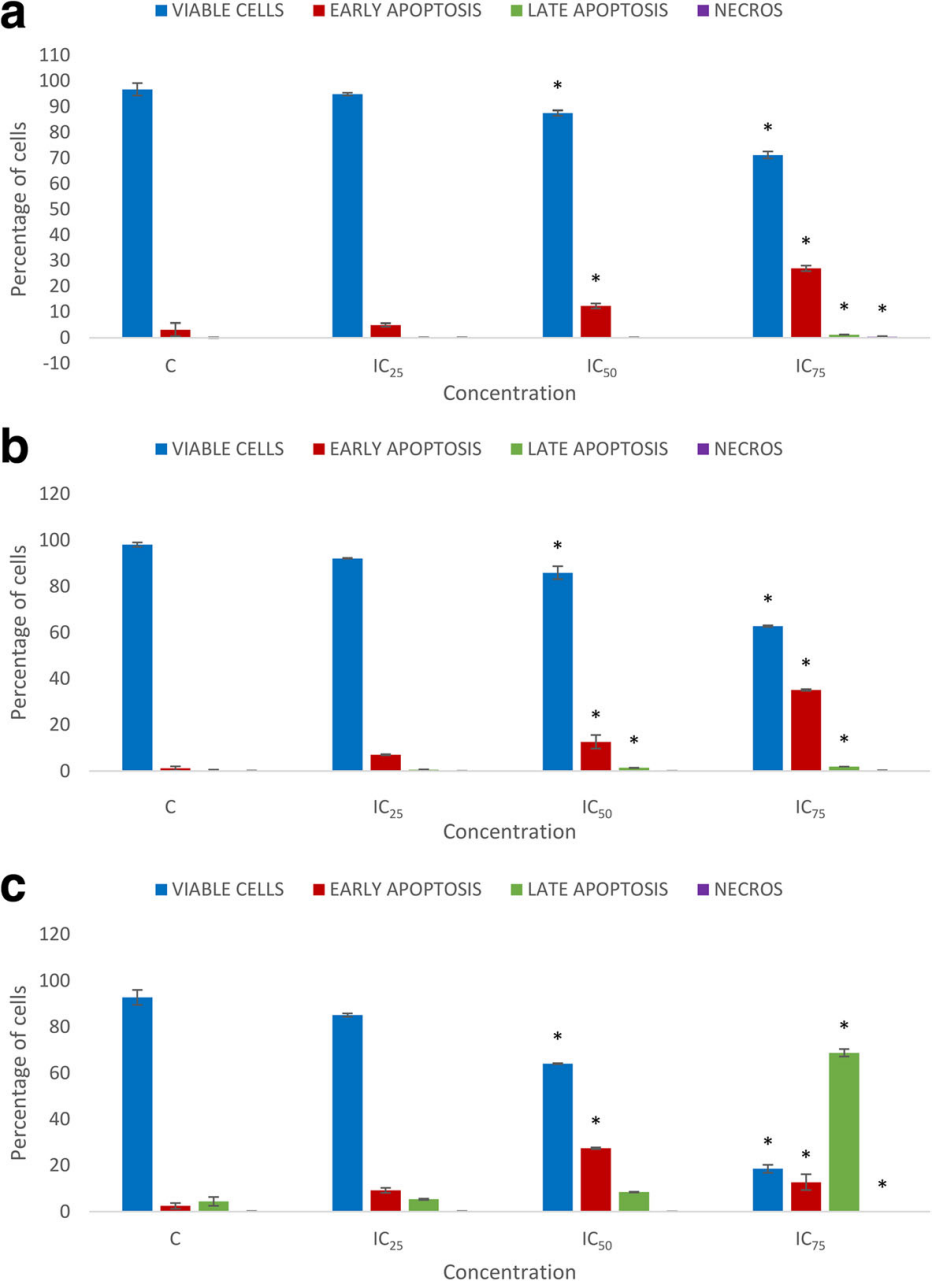
Effect of FAA on the induction of apoptosis in HepG2 cells. The proportion of cells present in early apoptosis, late apoptosis and necrosis stage after treatment by IC_25_ = 42, IC_50_ = 26.67 and IC_75_ = 11.67 of FAA for (**a**) 24, (**b**) 48 and (**c**) 72 h. Values are presented as means (*n* = 3) ± S.E. *signified (*p* < 0.05) compare to the control

Externalization of phosphatidylserine on the cell membrane is a distinctive feature of apoptotic cells. Annexin V is a recombinant protein which has a high affinity for this externalized moiety and can be used to detect apoptosis [33]. Apoptotic cell death was thus evaluated using annexin V-PI dual staining. Data analysis of HepG2 cell lines revealed that IC_50_ and IC_75_ values of FAA led to significant time- and dose-dependent reduction in the viable cell proportion. At 24 h of treatment, entering into the early apoptosis had sharply enhanced to 12.3% and 26.95% after treatment with IC_50_ and IC_75_ of FAA, compared to 3.2% in the control while the cell proportion present in late apoptosis was greatly raised to 1.25% by IC_75_ of FAA from 0.1% in the control. The cell percentage in necrosis stages after treatment with IC_75_ of FAA markedly increased to 0.5% from 0% in untreated cells. At 48 h, the percentages of cells entering the early apoptosis stage were significantly higher after treatment with IC_50_ and IC_75_ of FAA (12.5% and 35%), when compared to untreated cells (1.25%). Late apoptotic cell proportion also increased to 1.45% and 1.9% by IC_50_ and IC_75_ of FAA, compared to the untreated cells (0.45%). No significant effect was recorded in the percentages of necrotic cells at 48 h. In addition, 72 h treatment illustrated a dramatic increment in the percentage of early apoptotic cells treated with IC_50_ and IC_75_ of FAA (27.35%, 12.75%), compared to the untreated cells (2.5%). In contrast to 24 and 48 h, at 72 h, the percentage of late apoptotic cells was higher than early apoptosis, as late apoptotic cell percentage was dramatically enhanced by IC_50_ and IC_75_ of FAA (8.5% and 68.75%), compared with the untreated cells (4.45%). In contrast, the percentage of necrotic cells with IC_75_ of FAA decreased to 0% from 5.4% in the untreated cells (Fig. 5).

### Caspase-3 activity

**Fig. 6:**
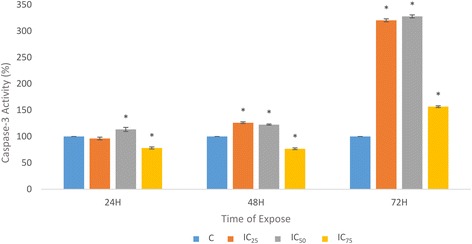
Effect of FAA on executioner caspases-3 activity in HepG2 cells. The cells were treated with IC_25_ = 42, IC_50_ = 26.67 and IC_75_ = 11.67 of FAA 24, 48 and 72 h treatment. Results are expressed as the mean optical density (405 nm) ± SD of three independent experiments. The symbol * indicates significant difference from control (*p* < 0.05)

The activity of caspase-3, the terminal effector in the apoptotic cascade and the enzymatic major marker of apoptosis, was assessed in a time- and dose-course manner. In the treated HepG2, the caspase-3 activity after 24 h increased only in IC_50_ of FAA. The increment reached a higher amount when the cells were treated with IC_25_ and IC_50_ of FAA at 48 h. Interestingly, with the increase of exposure time with FAA, caspase-3 activity had an eminent increase in all concentrations at 72 h compared to those values in the cells that were treated for 24 h and 48 h (Fig. 6).

### Expression level of corresponding genes in HepG2 cells

**Fig. 7:**
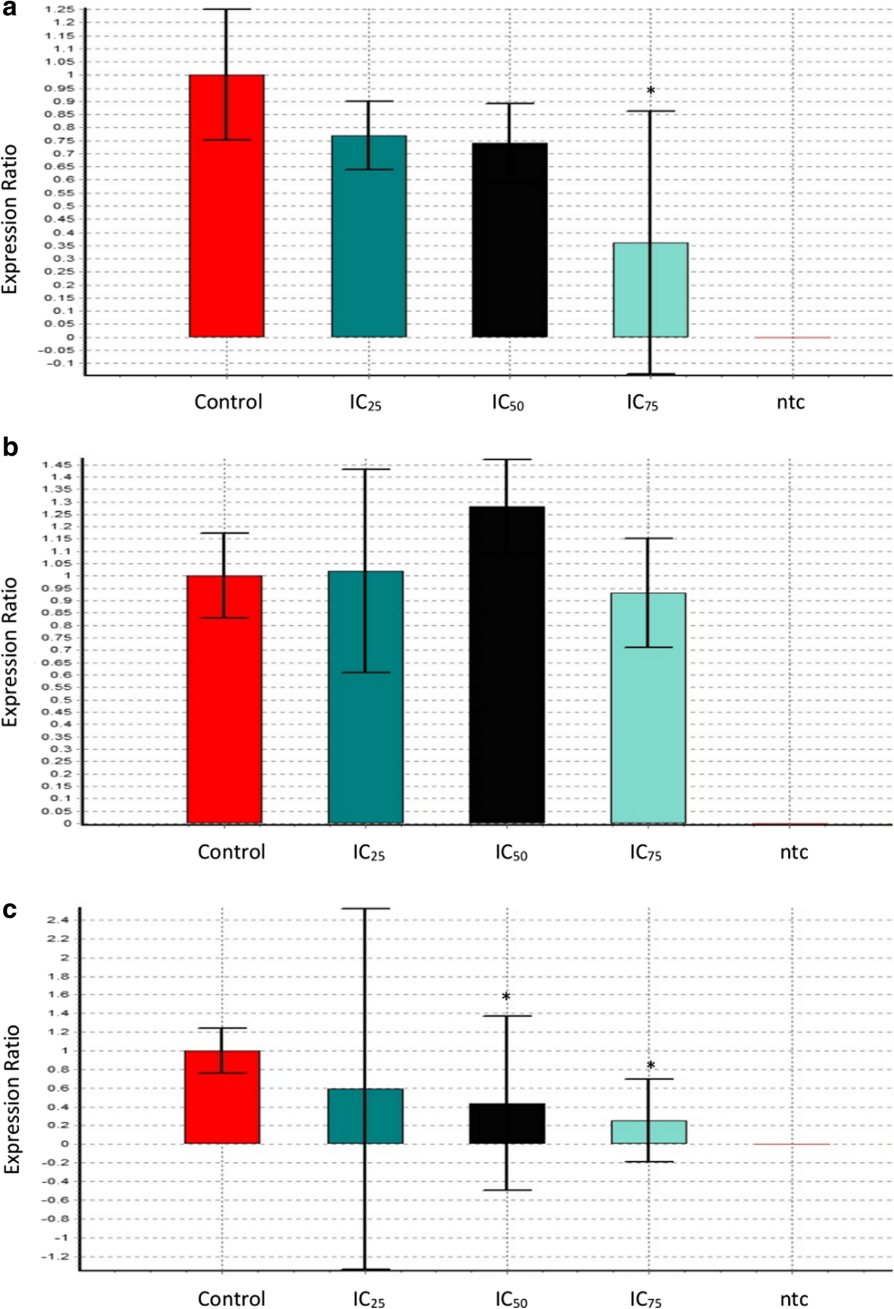
Real-time quantitative PCR analysis illustrates the gene expression in HepG2 cells. The relative quantification of the target gene, (**a**) *Bcl-2*, (**b**) *Cdk1* and (**c**) *p53*, by the delta–delta–Ct method was done using the Qiagen software after treatment with IC_25_ = 42, IC_50_ = 26.67 and IC_75_ = 11.67 of FAA for 24 h. A sample without cDNA template (ntc) was used as the negative control. Values are means ± SD of three independent experiments

The association between cell cycle and apoptosis related gene expression level in treated cells with the results obtained by proliferation and apoptosis tests were assessed by qPCR method. Evaluation of *Bcl-2*, *Cdk1* and *p53* expressions in FAA-treated HepG2 cells revealed alternative modulations in the level of expression. FAA treatment showed a slight (1.3-fold and 1.34-fold) downregulation of *Bcl-2* in IC_25_ and IC_50_ and a considerable (2.76 fold) downregulation in IC_75_. Regarding the expression levels of *Cdk1*, no significant alteration was found upon treatment with FAA, apart from a slight increase of marginal statistical significance after treatment with IC_25_ and IC_50_ by 1.02- and 1.28-fold. The *p53* was found to be downregulated after all three indicated concentrations of FAA, exhibiting a 1.69-, 2.26- and 3.94-fold decrease after treatment with IC_25_, IC_50_ and IC_75_ concentrations, respectively (Fig. 7).

### Synergistic effect

**Fig. 8:**
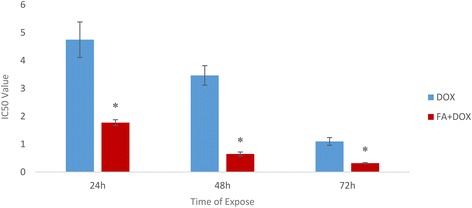
Interaction between doxorubicin and FAA in human liver cancer cell (HepG2). Values are calculated from three independent experiments. * = *p* < 0.05 compare the combination of doxorubicin-FAA and doxorubicin alone

**Fig. 9:**
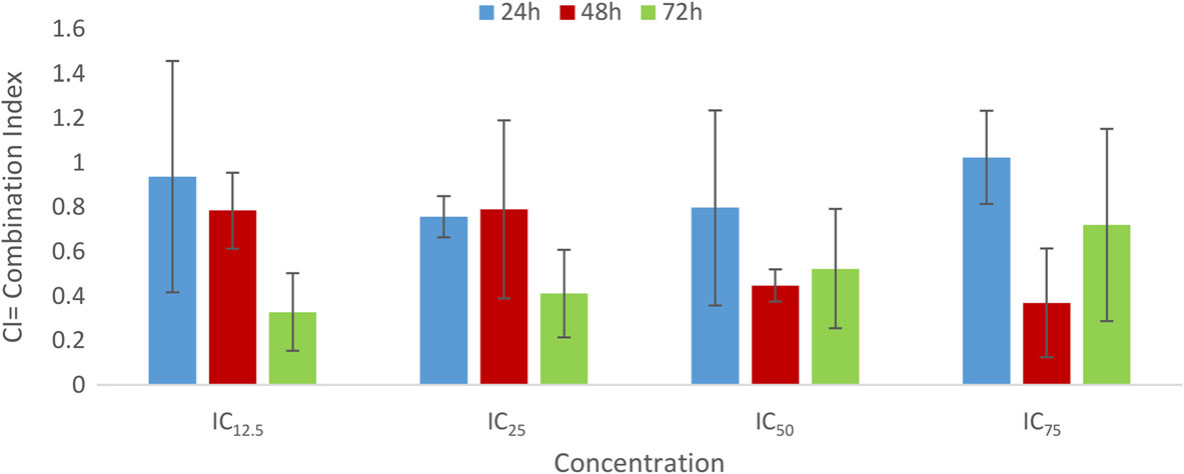
Synergistic effect of drug combination. Hepg2 Cells were treated with FAA-doxorubicin in a serial dilution for 24, 48 and 72 h. CI value was determined by Chou and Talalay method

In order to determine the FAA synergistic effect with doxorubicin, the concentrations of the extract and chemotherapeutic drug at their respective IC_50_ values against HepG2 cells were applied using an MTT assay. The mixture significantly inhibited the cell proliferation in a dose- and time-dependent manner, compared to the results of single agents. The IC_50_ of doxorubicin decreased considerably to 1.77 μg/ml, 0.65 μg/ml and 0.32 μg/ml at 24, 48 and 72 h (from 4.75, 3.47 and 1.7) (Fig. 8). Moreover, combination analysis was performed using the method described by Chou and Talalay through calculation of CI (combination index) [34], using CompuSyn software to assess the synergistic effect (CI < 1), additive effect (CI = 1), or antagonistic effect (CI > 1). The CI value indicated a synergistic effect in all concentrations and time periods, except for IC_75_ at 24 h (CI = 1.02, additive). The CI value showed an upward trend at 24 h (CI = 0.93, 0.75, 0.79, 1.02) and 72 h (CI = 0.32, 0.41, 0.52, 0.72), in IC_12.5_, IC_25_, IC_50_ and IC_75_, respectively, but a downward trend at 48 h (CI = 0.78, 0.79, 0.44, 0.37). The results demonstrated that the growth of HepG2 was inhibited significantly when doxorubicin was combined with FAA, as opposed to a single-agent treatment (Fig. 9).

## Discussion

Natural products and pharmaceutical compounds have been studied as cancer chemo-preventive agents, in vitro and in vivo [35]. There is limited information on the medicinal value of *Allium atroviolaceum*, particularly its cytotoxicity against cancer cells. Hence, the aim of the current study was evaluation of FAA extract potency on cell proliferation inhibition and inducing of apoptosis, which could contribute to a better understanding of the mechanisms of their potential carcinogenicity.

The present study demonstrated that FAA was an effective inhibitor against HepG2 cell proliferation, suggesting the presence of bioactive compounds in the extract [36]. However, the positive control, doxorubicin, showed a considerably stronger effect in comparison to FAA. Furthermore, the extract exhibited stronger cytotoxic activity after 72 h of exposure only at higher concentrations, whereas cytotoxic effects of lower concentrations were not significantly different from 24 and 48 h. This result indicates that the cells could be killed immediately after treatment by low concentrations, but after a long time, the surviving cells were stimulated for proliferation, or cells had adapted to the treatment and were recovered while the high FAA dose inhibits the proliferation in time course. In reality, cancer cells that exhibit resistance at one concentration may indicate growth inhibition at higher doses of the same preparation [37]. In addition, the applicability of a substance as a pharmacological drug depends on the balance between its therapeutic and toxicological effects [38]. The results showed that the extracts did not produce a cytotoxic effect towards normal cells, suggesting that the anticancer activity of FAA might be specific to HepG2 cells, in contrast to doxorubicin which was toxic against normal cells.

The suppression of cancer cell growth may occur through interference with fundamental cellular functions including apoptosis [39]. The difference between cytotoxicity and apoptosis is demonstrated by a series of specific morphological features [40]. One of the best methods for apoptosis definition is microscopic observation of cell morphology [41]. The morphology of treated cells demonstrates a substantial amount of cells undergoing apoptosis. The changes in cell shape after 24 h were heralded by cytoplasm condensation together with blebbing. Apoptosis proceeded to a subsequent stage after 48 h characterized by a rounding of cells and the frequent protrusion of apoptotic bodies. Cell necrosis was observed at 72 h, distinguished by initial cell swelling and bursting of both the endoplasmic reticulum and lysosomes. The latter of these two organelles contains digestive enzymes which contribute to further autolysis of the cell and its final disintegration. This releases cellular debris which elicits the inflammatory response that normally accompanies necrosis [42]. The results illustrated that apoptotic events may occur in different time courses that may depend on the inducing mechanism.

A comparison of nuclear morphology at these various stages by AO/PI stain using fluorescent microscopy suggested that the FAA treated HepG2 cells displayed nuclear morphological changes. Untreated cells were observed with a green intact nuclear structure whereby, early apoptosis is obvious by intercalated AO within the fragmented DNA. The features of early apoptotic death with nuclear chromatin condensation and margination were clearly observed at 24 h. While blebbing was noticed as moderate apoptosis after 48 h treatment with FAA. Blebbing is a normal cellular activity observed during mitosis. In damaged cells, the presence of blebs illustrates impending cell death and apoptotic cells unable to stop blebbing and flatten back onto the substratum [43, 44]. In addition, late stages of apoptosis were observed after 72 h treatment with FAA. Comparison of the nuclear and surface morphological changes indicated simultaneous events, regarding the time of active surface blebbing, shrinkage and formation of apoptotic bodies concomitant with nuclear margination during the early apoptotic process (24 h) and the formation of debris on the surface aligned with membrane permeability to PI in the necrosis process (72 h).

Based on the antiproliferative activity of FAA on HepG2 cells, the proportion of the cell in different phases of the cell cycle was analysed to determine the alteration of cell cycle phases, affected by FAA. The results exhibited a high proportion of cells at sub-G0 phase, because of nuclear DNA cleavage into multiple fragments [45], illustrating induced apoptosis cell death [46]. Controversially, the proportion of cells in the phase of G0/G1, S and G2/M (except IC_25_ of 48 h) considerably decreased in a dose-dependent manner compared to the control (Fig. 4). This result provided evidence that FAA induces apoptosis and, in turn, inhibits cell growth. Thus, apoptotic cell death of FAA was further analysed in HepG2 cells.

Apoptosis compared to necrosis is a desired somatic defence mechanism against cancer cells [41]. Annexin V/PI staining was performed producing a significant increase in the early- and late-apoptotic populations in various time- and dose-course in HepG2 cells. Annexin V- PI- considered as viable cells, while Annexin V+/PI- staining patterns showed early apoptotic cells; whereas Annexin V+/PI+ exhibited late apoptotic cells due to a loss of plasma membrane integrity [47] and VFITC-/PI+ was considered as necrotic cells [48]. In early apoptotic cells (24 and 48 h) the membrane integrity is retained, which is aligned with our observation by inverted and fluorescent microscopy, while late apoptotic cells with compromised membranes performed for a longer time (72 h) in treated cells with high concentration (IC_75_). This result demonstrated that entire time span for apoptosis, from early to the late apoptosis, is a very long process in HepG2 cells, exposed to FAA. It is worth noting that FAA did not promote necrosis at the times and dose tested in HepG2 cell line that showed relatively low percentages of annexin V−/PI+ cells over dose and time course possibly because of the release of pro-inflammatory intracellular contents [49], while the microscopic observation showed PI stained cells, displaying occurrence of necrosis. Apparently the membrane non-permeability of apoptotic cells could be kept for macromolecules temporarily, even after they become permeable for small charged molecules like PI, therefore, the time required for their elimination can be extended [50].

A major part of apoptosis could be mediated by caspase-3, the promoter and the terminal effector in the apoptotic cascade [41, 51]. The findings confirmed that FAA is capable to induce caspase-dependent apoptosis in a time-dependent manner. Cleavage of caspase-3 led to exposure of phosphatidylserine on the external surface of the plasma membrane, measureable by annexin V binding [52]. These results are in agreement with the obtained effect of FAA on apoptosis (Fig. 5), where the cells exposed to the lower concentrations (IC_25_ and IC_50_), showed an increase in caspase-3 activity, whereas IC_75_ concentration of FAA ended up with necrosis which is annexin V–. Moreover, the cleavage of caspase-3 accelerates disassembly of cells, including DNA fragmentation, chromatin condensation, nuclear remodelling and membrane blebbing, as detected in the morphological study which suggested the caspase mediated apoptosis in HepG2 cells [53–55]. It has been reported that caspase-3 is essential for cleavage of multiple protein substrates, including *Bcl-2* [56]. The expression level of *Bcl-2* anti-apoptotic gene illustrated a significant downregulation after treatment with IC_75_ of FAA. Recent evidence suggests that *Bcl-2* also acts as a downstream death substrate of caspases and, thus, the caspase enzymes may be able to deactivate the *Bcl-2* anti-apoptotic function and further enhance cell death, even when apoptosis is triggered via a non-*Bcl-2* dependent pathway. Although there is a feedback loop between *Bcl-2* and caspase, *Bcl-2* cannot always inhibit apoptosis, implying a subset of caspase activation that is likely to run through the death receptor pathway (not mitochondrial pathway) [57] that might occur in cells treated with lower concentrations. On the other hand, defective checkpoints are a feature of the majority of human cancers. Investigation into the expression level of *Cdk1* revealed no significant change in this gene that was aligned with the result of the cell cycle (Fig. 4).

Deficiencies of checkpoint are mainly affected by mutations of *p53* in many cancers [58]. The functional *p53* encodes a nuclear phosphoprotein that regulates the synthesis of gene products involved in growth arrest, DNA repair, apoptosis and the inhibition of angiogenesis [59]. According to the present results, FAA is not enabled to increase the *p53* level, even the expression was significantly downregulated. It could be concluded that FAA induces apoptosis in a *p53* independent pathway. Although usual induction of cell death needs *p53*, a reduced or delayed response can be activated via *p53*-independent mechanisms following DNA damage. Cancer cells lacking *p53* required signalling for cell cycle arrest; the absence of this response activates caspase-3 and mitotic catastrophe [60].

Moreover, FAA synergistically increases the inhibitory effect of doxorubicin on HepG2 cell growth compared with individual doxorubicin treatments. Further, the CI analysis of FAA-doxorubicin revealed dramatic synergistic cytotoxic effects. The CI values obtained for cancer cells were <1, confirming a synergistic interaction between combined treatments. The importance of this finding lies in the fact that although doxorubicin is a potent anticancer agent, no one can deny its hazardous toxicity against normal cells, the harmful side effects on health and the development of primary and secondary drug resistance in cancer [61]. The mechanism of action is unclear and, possibly, multiple compounds in the herbal extract or multiple pathways are involved. For instance, mitochondrial permeability transition increased and caspase-3 activation was delayed in an exposure of doxorubicin, while oxidative DNA damage was induced by the H_2_O_2_ generation which caused a Dox-induced apoptotic pathway [62]. Doxorubicin is a DNA-damaging agent that causes early activation of *p53* in tumour cells. DNA damage, allowing *p53* to function as a transcription factor inducing apoptosis, decreased *Bcl-2* expression and increased cell permeability, and subsequently activated caspase-3 [63, 64]. On the other hand, sensitivity of doxorubicin-mediated apoptotic signalling may be enhanced by FAA through *Bcl-2* downregulation via caspase-3 activation. Taken together, these findings indicate that a simultaneous blockade of different growth factor-driven signal-transduction pathways might lead to a more substantial antitumor effect [34].

## Conclusion

Our findings support previous literature related to the pharmacological activities of different closely-related species of *Allium*. FAA demonstrated dose-dependent anti-proliferative properties in human hepatocarcinoma cells (HepG2) and pro-apoptotic properties independent of the *p53* status of the cells. We also observed decreased expression of the anti-apoptotic protein *Bcl-2* in HepG2 which promotes the release of cytochrome c and further leads to activation of effector caspases-3. The presence of a sub-G0 population in cell cycle progression of HepG2 cells and cleaved caspase-3 staining suggests induction of apoptosis. These results have important clinical implications as they have commendable anti-proliferative activities against human hepatocarcinoma, without harming the normal cells, and it can be considered as an effective adjuvant therapeutic agent after clinical trials. However, further study of gene expression in time course and study of more genes related to apoptosis will improve our findings and give useful results to find a novel drug.

## Abbreviations

AO: acridine orange
FAA: flower of *Allium atroviolaceum*
HCC: hepatocellular carcinoma
PI: propidium iodide
